# Posterior reversible encephalopathy syndrome with spinal cord involvement as the first presentation of lupus nephritis

**Published:** 2019-10-07

**Authors:** Ali Asghar Okhovat, Siamak Abdi, Farzad Fatehi

**Affiliations:** 1 Department of Neurology, Shariati Hospital, Tehran University of Medical Sciences, Tehran, Iran; 2 Aix Marseille University, CNRS (UMR 7339), Centre de Resonance Magnetique Biologique et Medicale, Faculte de Medecine, Marseille, France

**Keywords:** Posterior Reversible Encephalopathy Syndrome, Spinal Cord, Systemic Lupus Erythematosus, Seizures

A 23-year-old woman was admitted to the emergency department with the history of headache, serial seizures, and decreased the level of consciousness from a week before. At admission, blood pressure was 230/170 mmHg, and creatinine level was 7.6 mg/dl. Initial brain and cervical magnetic resonance imaging (MRI) revealed hyperintense lesions on fluid-attenuated inversion recovery (FLAIR) in bilateral occipital lobes and a longitudinally extensive lesion in the spinal cord (Figure 1, A-C). In the laboratory investigations, the level of anti-double stranded DNA was 45 IU/ml (normal < 10 IU/ml) and anti-nuclear antibody titer was high (> 1/160). Moreover, in renal biopsy, lupus nephritis was reported. Two weeks later, after hypertension treatment, the hyperintense signals wholly disappeared (Figure 1, D-F).

**Figure 1:**
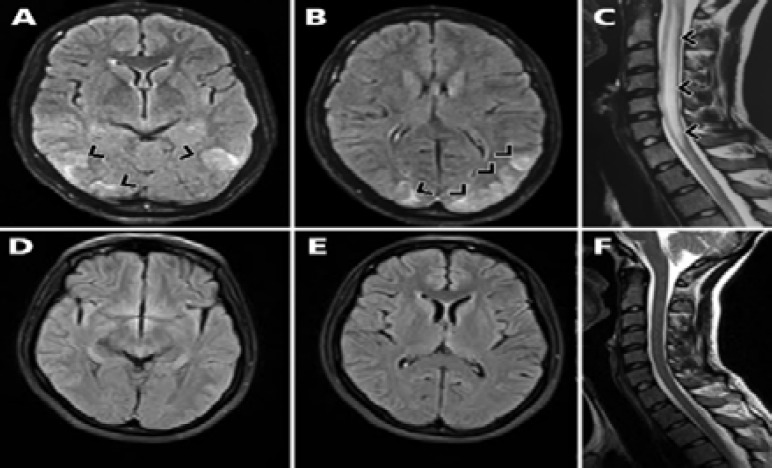
Axial fluid-attenuated inversion recovery (FLAIR) brain magnetic resonance imaging (MRI) indicating hypersignal lesions in parieto-occipital areas in favor of posterior reversible encephalopathy syndrome (PRES) at admission (A, B); sagittal T2 cervical MRI demonstrating a longitudinally extensive lesion in the spinal cord at admission (C); two weeks later, the hyperintense signals had completely disappeared on the brain and spinal cord MRIs (D-F).

Posterior reversible encephalopathy syndrome with spinal cord involvement (PRES-SCI (is a rare syndrome, which has not been reported as the primary manifestation of lupus nephritis. It is a syndrome manifested by characteristic clinical and radiological features. Clinical findings include headaches, nausea/vomiting, visual changes, altered consciousness, seizures, and focal neurological deficits.^1^^-^^3^  The MRI signal abnormality in classic PRES is located in the parieto-occipital region (consistent with vasogenic edema); but it may also affect the frontal and temporal lobes, basal ganglia, cerebellum, and brain stem.^   4 ^ PRES-SCI is an extremely rare syndrome that has been described recently.^3^^,^^5^  According to de Havenon et al., some of the PRES-SCI features may be unique including younger age of onset, higher occurrence in men, manifestation with acute hypertension crisis, headache, nausea/vomiting, encephalopathy, visual disturbances, renal failure, and hypertensive retinopathy.^   5 ^ Recently, a 42-year-old man has been described with uncontrolled hypertension and concomitant radiological features of PRES with sole involvement of the brain stem, cerebellum, and spinal cord.^   3 ^

The differential diagnosis of longitudinally extensive spinal T2 hyperintensity includes myelitis due to autoimmune diseases such as multiple sclerosis or neuromyelitis optica (NMO), central nervous system infections, malignancy, and myelopathies secondary to a dural arteriovenous fistula.^   1 ^ Nearly half of the patients with PRES have a history of autoimmune diseases such as systemic lupus erythematosus (SLE), rheumatoid arthritis, and Sjögren syndrome. In this patient, renal biopsy demonstrated proliferative glomerulonephritis due to SLE.

An interesting point in our patient was the first presentation of SLE with acute PRES-SCI syndrome that has not been described previously. Given that the acute encephalopathy syndrome in PRES-SCI may be misleading with the symptoms of an active neuropsychiatric SLE, SLE should be considered in the differential diagnosis of PRES-SCI, and appropriate workups to find the underlying rheumatological causes should be conducted.

## Notes:

**How to cite this article:** Okhovat AA, Abdi S, Fatehi F. Posterior reversible encephalopathy syndrome with spinal cord involvement as the first presentation of lupus nephritis. Iran J Neurol 2019; 18(4): 179-80.
